# A Novel Serum 4-microRNA Signature for Lung Cancer Detection

**DOI:** 10.1038/srep12464

**Published:** 2015-07-23

**Authors:** Ernest Nadal, Anna Truini, Asuka Nakata, Jules Lin, Rishindra M. Reddy, Andrew C. Chang, Nithya Ramnath, Noriko Gotoh, David G. Beer, Guoan Chen

**Affiliations:** 1 Section of Thoracic Surgery, Department of Surgery, University of Michigan Medical School, Ann Arbor, Michigan; 2 Department of Medical Oncology, Thoracic Oncology Multidisciplinary Unit, Catalan Institute of Oncology, L’Hospitalet, Barcelona, Spain; 3 Lung Cancer Unit, IRCCS AOU San Martino-IST National Institute for Cancer Research, Genova, Italy; 4 Division of Systems Biomedical Technology, Institute of Medical Science, University of Tokyo, Minato-ku, Tokyo, Japan; 5 Division of Hematology and Oncology, Department of Internal Medicine, University of Michigan Medical School, Ann Arbor, Michigan.

## Abstract

The aim of this study was to identify differentially-expressed miRNAs in the serum of non-small cell lung cancer (NSCLC) patients that might be a clinically-useful tool for lung cancer early detection. We performed miRNA expression profile analysis using TaqMan OpenArray Human panel in a discovery set of 70 serum samples obtained at lung tumor resection and 22 non-cancer subjects (NC). Selected serum miRNAs were then validated by quantitative PCR using an independent validation set of serum samples from LC patients (n = 84) and NC (n = 23). Sixty miRNAs were significantly up-regulated and 31 were down-regulated in the serum from NSCLC patients versus NC (adjusted *p* < 0.001). Four miRNAs (miR-193b, miR-301, miR-141 and miR-200b) were selected for validating their diagnostic value in an independent cohort. In the discovery set, the ROC plot derived from the combination of these miRNAs yielded an area under the curve (AUC) of 0.985 (95% CI 0.961–1.000, *p* < 0.001). In the test set, this miRNA signature exhibited an AUC of 0.993 (95% CI 0.979–1.000, *p* < 0.001). In conclusion, we identified a serum 4-miRNA signature that discriminated with high accuracy lung cancer patients from NC. Further prospective validation of this miRNA signature is warranted.

Lung cancer is the leading cause of cancer-related death worldwide with more than 200,000 new diagnoses and over 150,000 deaths expected to occur in 2014 in the United States^1^. Lung cancer is histologically classified as either small cell lung cancer (SCLC) or non–small cell lung cancer (NSCLC), with the latter accounting for over 80% of all lung cancers, including major subtypes such as lung adenocarcinoma (AC) and squamous cell carcinoma (SCC). Despite recent significant advances in the management of NSCLC and the use of targeted agents for specific molecular alterations, this disease has a poor 5-year survival rate (~15%), primarily attributable to late diagnosis when curative surgery is no longer an option. Early detection of lung cancer using low-dose CT has demonstrated in a large randomized trial a 20% reduction in mortality in heavy smokers as compared to chest X-rays^2^,^3^. However, this strategy has several limitations including high false-positive rates, potential over-diagnosis, excessive cost and the potential harm associated with radiation exposure. In this setting, the identification of non-invasive tumor biomarkers, able to detect the presence of malignancy or to predict tumor aggressiveness, might provide useful tools for earlier lung cancer diagnosis with the potential to reduce patient mortality.

MicroRNAs (miRNAs) are small noncoding RNAs involved in post-transcriptional regulation of gene expression by binding the 3′ untranslated region of their mRNA target transcripts. MiRNAs are released into blood circulation as a consequence of cell death or may also be actively secreted by cells^4^. Circulating miRNAs (c-miRNAs) might be an ideal class of biomarkers for blood-based cancer detection because: (i) miRNA expression is deregulated in cancer^5^,^6^; (ii) miRNA expression profiles are relatively cancer-specific^7^, (iii) miRNAs are stable in cell-free plasma or serum and thus readily detected by quantitative real-time PCR^8^,^9^;(iv) miRNAs may be more informative as upstream regulators of gene expression than mRNA classifiers^10^; (v) miRNAs may play a pathogenic role in the disease process by acting as oncogenes or tumor suppressor genes^11^,^12^. Multiple previous studies reported that c-miRNAs are able to distinguish with remarkable accuracy lung cancer patients from non-cancer subjects (NC)^9^,^13^,^14^. However, most c-miRNA signatures for lung cancer diagnosis included miRNAs overexpressed in blood cells which might have recapitulated the tumor-host interaction, but probably were not derived from the tumor. We hypothesized that up-regulated miRNAs in both NSCLC sera and tumors (based on our previous tumor miRNA profiling data in lung AC and SCC) that were not overexpressed in blood cells might be more lung cancer specific.

In this study we have profiled the miRNA expression of a large set of NSCLC and NC serum samples in order to identify c-miRNAs with potential utility for lung cancer diagnosis and prognosis, validating the diagnostic value of 4 miRNAs using an independent set of NSCLC and NC serum samples.

## Results

### Hierarchical clustering yielded two major clusters associated with clinical outcome and metastasis

**Figure 1:**
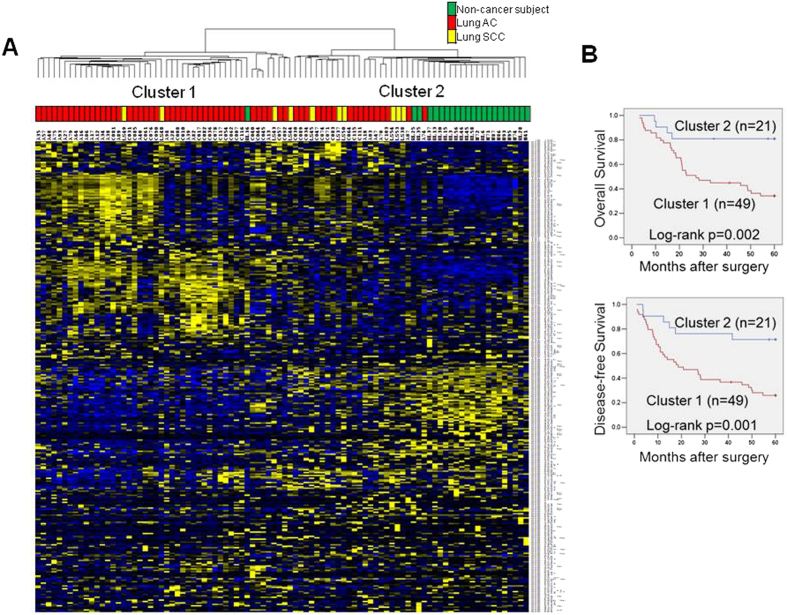
Hierarchical clustering analysis of serum miRNA expression. (**A**) Two major clusters were identified by unsupervised clustering analysis based on 334 expressed miRNAs in the serum of 70 NSCLC patients and 22 NC subjects. Samples are depicted in columns and microRNAs in rows. The histology of the samples (AC, adenocarcinoma; SCC, squamous cell carcinoma) is depicted in different colors at the top of the heat map. Overexpressed miRNAs are displayed in yellow whereas down-regulated microRNAs are displayed in blue. (**B**) Kaplan-Meier plot of overall survival and disease-free survival according to the cluster groups. Patient whose serum samples did not cluster with NC samples had significantly worse outcome.

Serum samples from NSCLC were randomly assigned to 2 independent sets (discovery and validation), consisting of 70 and 84 samples respectively. NC samples were selected to be age and gender-matched with NSCLC samples. Clinical and demographic characteristics of subjects included in both sets are provided in Table 1. In the discovery set, hierarchical clustering analysis based on the 334 expressed miRNAs revealed that most NC clustered together, whereas lung SCC and AC serum samples appeared more intermixed (Fig. 1A). We determined the correlation between these miRNA clusters and the clinical variables (Supplementary Table S1). There was no statistically significant difference in age, sex, stage or differentiation among these miRNA clusters. Interestingly, patients who did not cluster with NC had a significantly higher rate of recurrence (*p* < 0.049) and death (*p* = 0.001). Patients classified in cluster 1 had a significantly shorter OS and DFS (median: 27 and 18.6 months, respectively) as compared with cluster 2 (median: not reached for both, Log-rank test *p* = 0.002 and 0.001, respectively, Fig. 1B). In the multivariable analysis after adjusting by age, gender and disease stage, cluster 1 was independently associated with shorter OS (HR = 3.19, 95% CI 1.11–9.12, *p* = 0.031) and shorter DFS (HR = 2.89, 95% CI 1.19–7.01, *p* = 0.031).

**Figure 2:**
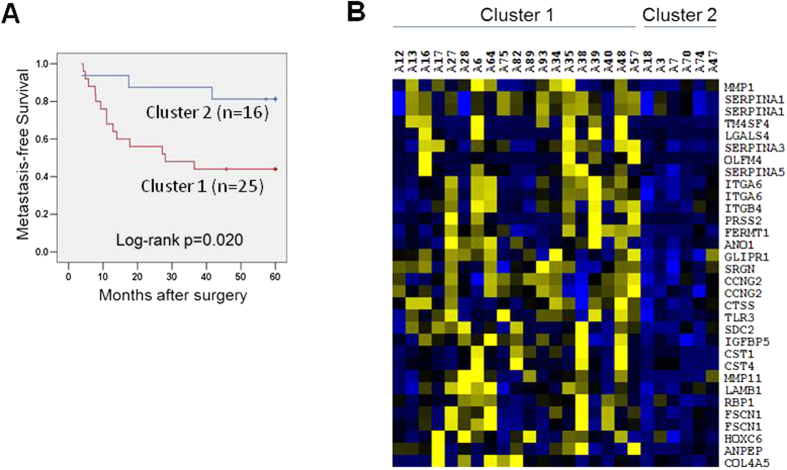
Relationship of serum miRNA profile and tumor metastasis. (**A**) Kaplan-Meier plot of metastasis-free survival (MFS) according to the cluster groups for stage I and II patients. Patients classified in cluster 1 had a significantly shorter MFS. (**B**) Heat map showing expression values of genes associated with metastasis in a subset of 25 lung AC. A significant number of genes associated with metastasis were overexpressed in tumors classified into the serum miRNA cluster 1.

In addition, stage I and II patients classified in cluster 1 had a significantly shorter metastasis-free survival (MFS, 28 months) as compared to cluster 2 patients (not reached, Log-rank *p* = 0.020, Fig. 2A). We compared the mRNA expression profile of 25 lung AC for which serum miRNA and tumor mRNA microarray data were available in order to identify differentially-expressed genes in the primary tumors included in cluster 1 (n = 19) versus tumors included in cluster 2 (n = 6). Interestingly, among the genes significantly overexpressed (*p* < 0.05, fold change >1.5) in cluster 1 tumors as compared to cluster 2, there were multiple genes that are involved in the acquisition of the metastatic phenotype such as serpins (*SERPINA1*, *SERPINA3* and *SERPINA5*), metalloproteinase inhibitors (*MMP1* and *MMP11*) and integrins (*ITG4* and *ITG6*) (Fig. 2B). Based on these results, we hypothesize that NSCLC with higher metastatic potential and more aggressive behavior have a different serum miRNA expression profile which might be explained by the plausible presence of circulating tumor cells in the bloodstream or a distinctive interaction between the primary tumor and the tumor microenvironment.

### Identification of differentially-expressed miRNAs in the serum of NSCLC patients versus NC subjects

A total of 91 miRNAs were differentially expressed in 70 NSCLC sera versus 22 age and gender-matched NC sera by unpaired class-comparison analysis (adjusted t-test *p* ≤ 0.001). Sixty were found significantly up-regulated (fold-change ≥1.20) and 31 were down-regulated (fold-change ≤ 0.60) in the NSCLC serum (Supplementary Table S2). Using miRNA expression data from our previous studies^10^,^15^, we determined whether these 91 significant miRNAs were also differentially expressed in primary tumors (lung AC and/or lung SCC) versus nonmalignant lung. Interestingly, only 24 out of 91 (26%) miRNAs were found deregulated in the same direction in the NSCLC tissues and serum, whereas 33 miRNAs out of 91 (36%) were deregulated in opposite directions in NSCLC serum and tissues (Supplementary Table S2). Moreover, there were 34 (37%) miRNAs that were not differentially expressed among the NSCLC and nonmalignant lung tissues or were not assessed in our previous studies.

The miRNAs overexpressed in both NSCLC tumors and sera are shown in Table 2. These miRNAs might be potentially useful diagnostic markers for lung cancer detection, as they are likely to be derived from the primary tumors or the circulating lung cancer cells. Only 5 miRNAs were found overexpressed in both lung AC and lung SCC primary tumors (miR-141, miR-200b, miR-193b, miR-200c and miR-106b) as well as in the NSCLC serum. Pathway enrichment analysis based upon the validated gene targets revealed that these 5 miRNAs were significantly associated (*p* < 0.001) with pathways relevant in lung cancer biology and lung development such as MAPK, PI3K-AKT, p53, ErbB, focal adhesion, steroid hormone biosynthesis, HIF1 and neurotrophin signaling pathways (Supplementary Table S3). Interestingly, miRNAs associated with miR-17 family (miR-17, miR-19a, miR-19b, miR-20b, miR-106a, miR-106b, miR-93, and miR-25) were overexpressed not only in the serum and the primary tumors from NSCLC patients, but also in the blood cells suggesting that these miRNAs might be reflecting the host response against the tumor.

### Identification and validation of 4-miRNA signature for lung cancer detection

**Figure 3:**
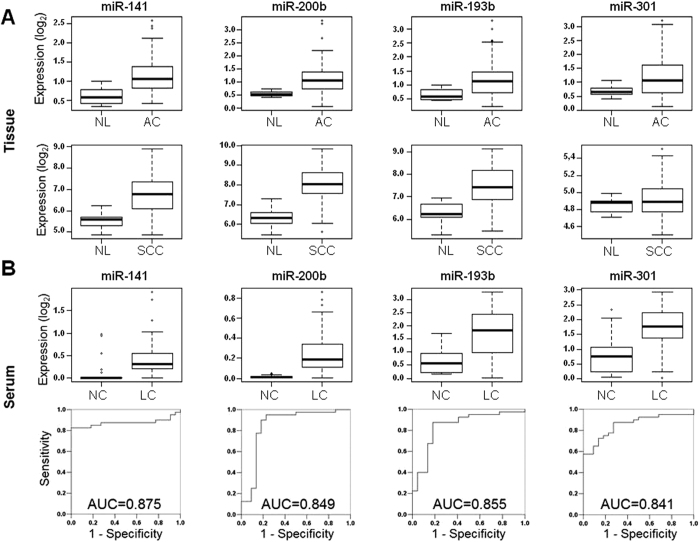
Expression level of selected miRNAs in lung tissues or serum samples. (**A**) Box plots representing microarray expression results for miR-141, miR-200b, miR-193b and miR-301 from two different studies performed in lung primary tumors. The upper panel shows the expression values for 10 nonmalignant lung samples (NL) and 91 lung adenocarcinomas (AC), whereas the bottom panel shows the expression values for 10 NL and 61 lung squamous cell carcinomas (SCC). The y-axis depicts log_2_ fold change. All four miRNAs were significantly overexpressed (*p* < 0.001) in lung tumors, except miR-301 that was not significantly higher in lung SCC. (**B**) Box plots showing the microarray relative expression of miR-141, miR-200b, miR-193b and miR-301 in the serum of 22 non-cancer subjects (NC) and 70 NSCLC patients (LC). The y-axis depicts log_2_ fold change. All four miRNAs were significantly overexpressed (*p* < 0.001) in the serum of LC patients as compared to NC. This expression pattern was concordant with the tumor miRNA profile. The ROC curves to detect stage I lung cancer are shown at the bottom for each miRNA. All 4 miRNAs yielded an AUC > 0.840.

To construct the diagnostic signature, the miRNA candidates were selected based upon the following criteria: miRNAs significantly up-regulated (adjusted t-test *p* < 0.001) in the NSCLC tissue and serum as compared to normal lung tissue and NC serum respectively, not overexpressed in circulating blood cells and with Area Under the Curve (AUC) > 0.840 for discriminating stage I LC from NC in the receiver-operating characteristic (ROC) plots. We identified 4 miRNAs that fulfilled these criteria: miR-141, miR-200b, miR-193b and miR-301, since they were significantly overexpressed in lung AC and/or SCC versus normal lung samples (Fig. 3A) as well as in the serum of NSCLC patients versus NC (Fig. 3B). Additionally, they yielded an optimal AUC for detecting stage I lung cancer and were expressed at relatively high levels by lung cancer cell lines (Supplementary Figure S2). We assessed the prognostic value of those 4 serum miRNAs, but their expression was not associated with clinical outcome (Supplementary Figure S3).

**Figure 4:**
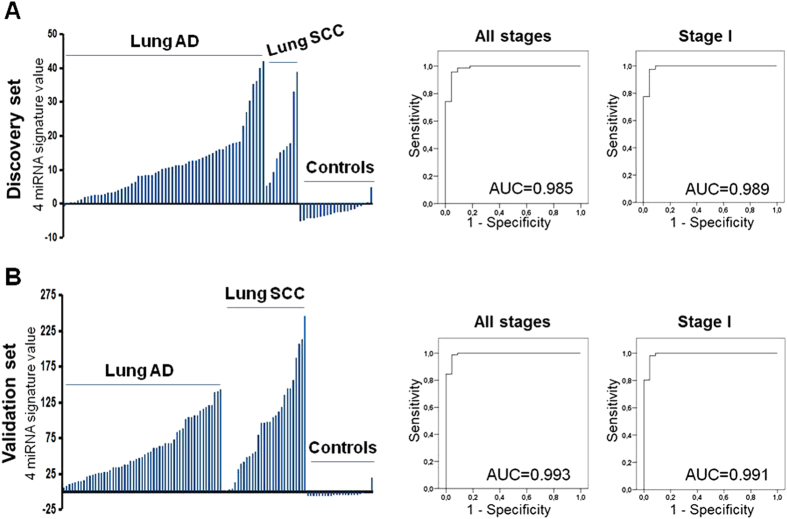
Discovery and validation of a 4-miRNA signature for lung cancer diagnosis. (**A**) Bar plots representing the expression values and ROC curves to detect lung cancer in the discovery set comprised by 60 AC, 10 SCC and 22 NC. (**B**) Bar plots representing the expression values and ROC curves to detect lung cancer in the validation set comprised by 58 AC, 29 SCC and 23 NC.

Next, a diagnostic signature was obtained by logistic regression based upon the expression values of these 4 serum miRNAs in the discovery set, and this miRNA signature generated an AUC of 0.985 (95% CI 0.961–1.000, *p* < 0.001) for detecting NSCLC (all stages) and of 0.989 (95% CI 0.967–1.000, *p* < 0.001) for detecting stage I NSCLC (Fig. 4A) in the discovery set. The optimal cut-off to distinguish NSCLC from NC subjects was set at 0.276 based upon the ROC plot. Using this threshold, the miRNA signature achieved a sensitivity of 96% and a specificity of 95%. To validate these findings, we measured by RT-PCR the expression level of miR-141, miR-200b, miR-193b and miR-301 in the serum of an independent cohort of 84 NSCLC and 23 age and sex-matched NC subjects. U6 snRNA was identified as a suitable reference miRNA in the discovery set and was used for data normalization in the test set. Validation results showed that all 4 miRNAs were significantly overexpressed in the NSCLC sera (*p* < 0.001) and the log_2_ fold-change for miR-141, miR-200b, miR-193b and miR-301 were 2.67, 2.98, 2.52 and 2.01 respectively. The combination of these 4 miRNAs yielded an AUC of 0.993 (95% CI 0.979–1.000, *p* < 0 .001) for detecting NSCLC (all stages) and of 0.991 (95% CI 0.973–1.000, *p* < 0.001) for detecting stage I NSCLC (Fig. 4B), supporting the diagnostic value of this serum miRNA signature. Using the logistic regression model obtained in the training set and fixing the cut-off that was defined in the training set, the sensitivity of this miRNA signature was 97% and the specificity of 96% in the validation set.

## Discussion

Numerous studies have shown that c-miRNAs are potentially useful diagnostic biomarkers for distinct human diseases. MiRNAs are released into the blood circulation as a result of apoptotic or necrotic cell death, but active secretion of miRNAs has been suggested as a mechanism of cell-to-cell communication as well as an alternative source of c-miRNAs^4^. C-miRNAs are highly stable, since they are incorporated into microvesicles (such as exosomes and apoptotic bodies) or bound to ribonucleoprotein complexes (such as argonaute 2, the effector component of the miRNA-silencing complex). However, c-miRNAs may arise from various cells, including normal blood cells as well as by cells associated with the tumor microenvironment (e.g. endothelial or immune cells). This makes identification of tumor specific cell-free miRNAs more challenging^16^,^17^. We hypothesized that NSCLC-specific miRNAs are likely to be coordinately deregulated in both NSCLC sera and tumors, and should not be overexpressed in blood cells.

In this study, the serum miRNAs from 70 NSCLC patients and 22 NC subjects were profiled using a PCR-based miRNA array. We found 91 miRNAs significantly deregulated (60 up- and 31 down-regulated) in the NSCLC serum versus NC. Based upon microarray miRNA data from our previous studies performed in NSCLC tumors, only few miRNAs appeared to be overexpressed in both tumors and serum. In accordance to previous investigations^13^,^18^, numerous miRNAs overexpressed in primary tumors were not detectable or deregulated in the NSCLC serum, further underscoring the complexity of defining tumor-specific miRNAs. Hierarchical clustering analysis based on 334 miRNAs yielded two major clusters of NSCLC, with most of cancer-free subjects clustering together within cluster 2. Although no statistically significant differences in age, sex, tumor stage or differentiation were found between these two clusters, patients classified in cluster 1 had poorer outcome as compared to cluster 2. When comparing mRNA expression data from a small subset of NSCLC tumors from cluster 1 and 2, genes associated with a metastatic phenotype were overexpressed in tumors from cluster 1. These results suggested that serum miRNA expression profiles might distinguish surgically-resected NSCLC with more aggressive disease and higher metastatic potential, although further validation is warranted.

A supervised diagnostic miRNA signature composed of 4 miRNAs (miR-141, miR-200b, miR-193b and miR-301) which were up-regulated in NSCLC tumors and serum was selected to validate its diagnostic value in an independent cohort of age and sex-matched serum samples. This serum miRNA signature distinguished accurately amongst NSCLC and cancer-free subjects, independent of tumor stage and tumor histology.

Two of the miRNAs included in the supervised miRNA signature are also up-regulated in the serum of patients diagnosed with other types of cancers, such as prostate (miR-141) and pancreatic cancer (miR-200b)^8^,^19^. MiR-301 was found overexpressed among vesicle-related miRNAs from plasma in NSCLC^20^, but it has not been previously reported as a serum-based marker for cancer diagnosis. MiR-193b has not been previously reported as blood-based marker for cancer diagnosis. Although the inclusion of miR-141 and miR-200b may reduce the specificity of the proposed miRNA signature, this signature discriminated between NSCLC patients and matched controls with outstanding accuracy in the validation set. We plan to validate the diagnostic utility of this miRNA signature using an independent cohort of serum samples obtained from subjects at high risk of lung cancer such as heavy smokers who are participating in a lung CT screening program.

In conclusion, a serum 4-miRNA signature that discriminated with high accuracy clinically important subtypes of NSCLC from cancer-free subjects was successfully identified and validated. Although the clinical utility of blood-based miRNA signatures is promising, further validation of this serum miRNA signature in additional prospective cohorts including more non-cancer controls is warranted.

## Material and Methods

### Clinical samples

We used serum samples from 154 NSCLC patients (including 115 lung AC and 39 lung SCC) who underwent tumor resection at the University of Michigan from 1991-2007 and 45 non-cancer subjects. The peripheral blood was drawn at the time of lung tumor resection for all NSCLC and none of subjects had received preoperative radiation or chemotherapy. The NC subjects consisted of healthy controls as well as patients with non-cancer lung diseases (mainly COPD, bronchiectasis and pneumonia) and their blood was drawn at the outpatient clinic. Written patient consent and approval of the Institutional Review Board of the University of Michigan Medical School were obtained to collect specimens from patients undergoing lobectomy for lung cancer at the University of Michigan Medical Center, Ann Arbor, MI. The blood was processed for serum extraction within 1 hour and then flash-frozen into liquid nitrogen for long-term storage at −80 °C. Clinical data were retrospectively collected from the medical records and all cases were staged according to the revised AJCC/UICC 7^th^ edition TNM classification schema^21^.

### miRNA and mRNA profiling data from lung primary tumors

In this study, we used miRNA expression data from two previous published studies in lung SCC and lung AC^10^,^15^. In the lung SCC study, 61 primary tumors and 10 matched nonmalignant lung samples were miRNA profiled using mirVana Bioarray chip (Ambion, version 2) which contains 328 human miRNA probes (GSE16025). In the lung AC study, 91 primary tumors and 10 matched nonmalignant lung samples were miRNA profiled using TaqMan OpenArray Human microRNA panel (Applied Biosystems) which includes 754 miRNAs. A subset of 25 lung AC tumors for which serum miRNA was profiled had Affymetrix U133A gene expression microarray data from a previous study^22^. Details on RNA extraction, array preparation and data normalization were provided in our previous publications.

### Serum RNA isolation, miRNA profiling and data normalization

Total RNA was isolated from 400 μl of serum using miRVana PARIS kit (Ambion), following the manufacturer’s protocol. RNA concentration was measured by Nanodrop 2000 spectrophotometer (Thermo Scientific) and stored at −80 °C. Whole-genome serum miRNA profiling was performed in 92 serum samples using TaqMan OpenArray Human microRNA panel (Applied Biosystems). Input serum RNA was reverse-transcribed using TaqMan MicroRNA Reverse Transcription Kit and the Megaplex RT Primers (Applied Biosystems) in a PTC-100 (MJ Research) with 40 cycles of 16 °C for 2 min, 42 °C for 1 min, 50 °C for 1 sec and then 85 °C for 5 min. Resultant cDNA was preamplified using Megaplex PreAmp Primers and TaqMan PreAmp Master Mix (2X) in a PTC-100 (MJ Research) at 95 °C for 10 min, 55 °C for 2 min, 72 °C for 2 min, followed by 12 cycles at 95 °C for 15 sec and 60 °C for 10 min. Preamplified cDNA quality was assessed by quantitative RT-PCR to measure U6snRNA. Next, preamplified cDNA was mixed with TaqMan OpenArray Real-Time PCR Master Mix and loaded onto the cards using theAccuFill™ System. The cards were cycled in an OpenArray NT Cycler System (Applied Biosystems) at the University of Michigan Array Core. Data were extracted using the OpenArray Real-Time qPCR Analysis software (Applied Biosystems) and missing values for each miRNA were filled with maximum Ct + 2. MiRNAs with more than 50% missing data across all NSCLC samples were filtered out and a total of 334 human miRNAs were retained in the final analysis. Since there is no consensus on the optimal housekeeping gene in serum, we used the average Ct for all miRNAs as a loading control for each sample. Fold-change was calculated using the 2^(−ΔΔCt)^ method^23^, and then data were log_2_-transformed. All methods were carried out in accordance with the approved guidelines.

### Validation of miRNA expression by quantitative RT-PCR

Quantitative RT-PCR (qRT-PCR) was performed using TaqMan microRNA assays (Applied Biosystems) to determine the serum expression values of 4 miRs in an independent cohort of 84 NSCLC and 23 NC subjects. cDNA was produced and preamplified as described above and following a 1:12 dilution, amplified in the presence of TaqMan Mastermix with specific TaqMan probes (Applied Biosystems), according to the manufacturer’s instructions. qRT-PCR was carried out on an Applied BioSystems 7900HT thermocycler at 95 °C for 10 min, followed by 40 cycles of 95 °C for 15 sec and 60 °C for 1 min. Data were analyzed with SDS Relative Quantification Software version 2.2.2 (Applied BioSystems), after setting a Ct threshold of 0.2 and a manual baseline from 3 to 18 cycles. All experiments were carried out in duplicate. As the variability of U6 snRNA expression was small in the training set, it was used as endogenous control in the validation set (Supplementary Figure S1) and fold-change was calculated using the 2^(−ΔΔCt)^ method and the data were log_2_-transformed.

### Statistical analysis

Unpaired class comparison analysis was performed among serum miRNA expression levels in NSCLC versus NC and p-value was adjusted for multiple comparisons using Benjamini-Hochberg method^24^. To identify miRNA expression patterns, an unsupervised hierarchical centroid linkage cluster analysis was performed after mean-centering miRNAs and arrays using Cluster v3.0 and heat maps were visualized using TreeView software^25^,^26^. DIANA-miRPath software version 2.0 was used for pathway enrichment for the top miRNAs differentially expressed in the serum of NSCLC patients and overexpressed in the NSCLC tumors^27^. A miRNA signature was obtained by performing binary logistic regression based upon the expression values of 4 selected miRNAs in the training set and was then applied to the validation set. Pearson’s Chi square and ANOVA tests were used to determine the correlation between the clusters and the clinicopathological variables. Survival curves were plotted using the Kaplan-Meier method and survival differences were assessed by the log-rank test. Multivariable Cox proportional hazards were calculated adjusting by age, sex and stage. Disease-free survival (DFS) was measured from the date of surgery to the time of recurrence, death, or censoring. Overall survival (OS) was measured from date of surgery to the time of death or censoring.

## Additional Information

**How to cite this article**: Nadal, E. *et al.* A Novel Serum 4-microRNA Signature for Lung Cancer Detection. *Sci. Rep.* **5**, 12464; doi: 10.1038/srep12464 (2015).

## Supplementary Material

Supplementary Information

Supplementary Data

## Figures and Tables

**Table 1:** Characteristics of subjects in the discovery and validation set.

	**Discovery set**			**Validation set**		
	**NSCLC n = 70**	**Control n = 22**	***p*-value**	**NSCLC n = 84**	**Control n = 23**	***p*-value**
**Age, median**	67.5	67.0	0.144	65.5	60	0.074
**Gender, n (%)**						
Male	35 (50%)	13 (54%)	0.809	43(51%)	8 (35%)	0.239
Female	35 (50%)	9 (46%)		41 (49%)	15 (65%)	
**Smoking history, n (%)**						
Smoker	59 (84%)	6 (27%)	<0.05	79 (94%)	11 (48%)	<0.05
Nonsmoker	10 (14%)	8 (36.5%)		5 (6%)	6 (26%)	
Unknown	1 (2%)	8 (36.5%)		0 (0%)	6 (26%)	
**Stage, n (%)**						
I	40 (57%)	NA	NA	56 (67%)	NA	NA
II	18 (26%)	NA		19 (22%)	NA	
III	12 (17%)	NA		9 (11%)	NA	
**Histology, n (%)**						
Adenocarcinoma	60 (86%)	NA	NA	55 (65.5%)	NA	NA
Squamous carcinoma	10 (14%)	NA		29 (34.5%)	NA	
**Type of surgery, n (%)**						
Segmentectomy	3 (4%)	NA	NA	5 (6%)	NA	NA
Lobectomy	61 (87%)	NA		73 (87%)	NA	
Bilobectomy	2 (3%)	NA		3 (3.5%)	NA	
Pneumonectomy	4 (6%)	NA		3 (3.5%)	NA	

NA: not assessed.

**Table 2:** List of serum miRNAs significantly (*p* < 0.001) overexpressed in NSCLC versus NC that were up-regulated as well in lung adenocarcinoma (AC) or squamous cell carcinoma (SCC) primary tumors as compare to nonmalignant lung tissue (NL).

**miRNA**	**AUC stage I**	**Serum NSCLC/NC**	**Lung AC/NL**	**Lung SCC/NL**	**High expression in blood cells**	**Reported in NSCLC**	**Reported in other cancers**
**miR-141**	0.875	up	up	up	no	no	18663219; 23935962
**miR-193b**	0.855	up	up	up	NA	no	24778027
**miR-200b**	0.849	up	up	up	no	no	23272653; 20551052
**miR-301**	0.841	up	up	NS	NA	20595154	no
let-7g	0.840	up	NS	up	NA	no	24709885
miR-331	0.748	up	NS	up	NA	no	21035526
miR-758	0.747	up	up	NA	NA	no	no
miR-744	0.726	up	up	NA	NA	no	22432036
miR-106a	0.958	up	NS	up	yes	21544802	20234369; 25140035
miR-19a	0.948	up	up	NS	yes	no	no
miR-17	0.918	up	NS	up	yes	no	23056289
miR-19b	0.916	up	NS	up	yes	no	23874370; 24498016
miR-93	0.899	up	NS	up	yes	no	23748853; 24498016
miR-20b	0.875	up	NS	up	yes	20595154	no
miR-106b	0.838	up	up	up	yes	no	20234369; 23874370
miR-215	0.828	up	up	NS	yes	no	22353773; 24993656
miR-25	0.826	up	NS	up	yes	no	24595006; 24651474
miR-200c	0.820	up	up	up	yes	no	22954417; 23272653
miR-93*	0.739	up	up	NA	yes	no	no
miR-24	0.715	up	NS	up	yes	no	23697990

The AUC for detecting stage I NSCLC was calculated for each individual miRNA. Most of these miRNAs were overexpressed in blood cells according to the literature and reported as circulating diagnostic markers in other human cancers. PubMed IDs (PMID) were provided. NA: not assessed; NS: not significant.
