# Supplementary material for: A Novel Serum 4-microRNA Signature for Lung Cancer Detection
Source: Sci Rep. 2015 Jul 23;5:12464. doi: 10.1038/srep12464 (PMC5378885; doi:10.1038/srep12464)

**Supplementary Tables.**

**A Novel Serum 4-microRNA Signature for Lung Cancer Detection**

Ernest Nadal M.D., Anna Truini M.S., Asuka Nakata Ph.D., Jules Lin M.D., Rishindra M. Reddy M.D., Andrew C. Chang M.D., Nithya Ramnath M.D., Noriko Gotoh Ph.D., David G. Beer Ph.D. and Guoan Chen M.D., Ph.D.

**Supplementary Table S1.** Correlation between clinical variables and miRNA clusters identified by hierarchical cluster analysis. P-values were computed by using Fisher’s exact test or Chi-square test.

| **Patient characteristics** | **Cluster 1** | **Cluster 2** |  |
| --- | --- | --- | --- |
|  | **n=49** | **n=21** | ***p*-value** |
| **Age, median** | 69 | 65 |  |
| **Gender, n (%)** |  |  |  |
| Male | 27 (55%) | 8 (38%) |  |
| Female | 22 (45%) | 13 (62%) | 0.297 |
| **Smoking, n (%)** |  |  |  |
| Current | 6 (12%) | 2 (9.5%) |  |
| Former | 35 (71%) | 17 (81%) |  |
| Nonsmoker | 7 (14%) | 2 (9.5%) |  |
| Unknown | 1 (2%) | 0 (0%) | 0.810 |
| **Stage, (%)** |  |  |  |
| Stage I | 26 (53%) | 14 (67%) |  |
| Stage II | 13 (26.5%) | 5 (24%) |  |
| Stage III | 10 (20.5%) | 2 (9.5%) | 0.464 |
| **Histology, n (%)** |  |  |  |
| Adenocarcinoma | 44 (90%) | 16 (76%) |  |
| Squamous cell carcinoma | 5 (10%) | 5 (24%) | 0.154 |
| **Differentiation, n (%)** |  |  |  |
| Well | 9 (19%) | 5 (26%) |  |
| Moderate | 19 (40%) | 5 (26%) |  |
| Poor | 20 (42%) | 9 (48%) | 0.564 |
| **Recurrence, n (%)** |  |  |  |
| Yes | 26 (59%) | 5 (28%) |  |
| No | 18 (41%) | 13 (72%) | **0.049** |
| **Death, n (%)** |  |  |  |
| Yes | 32 (65%) | 4 (19%) |  |
| No | 17 (35%) | 17 (81%) | **0.001** |

**Supplementary Table S2.** List of differentially expressed miRNAs in the NSCLC serum as compared to NC. Student's t-test p-value among NSCLC and NC was adjusted by using Benjamini-Hochberg method.

|  | **Average** | **Average** | **Adjusted** | **NSCLC/NC** | **lung AC/NL** | **lung SCC/NL** |
| --- | --- | --- | --- | --- | --- | --- |
| **miRNA** | **NSCLC** | **NC** | ***p*-value** | **serum** | **tissue** | **tissue** |
| miR-125b | 1.91 | 0.39 | 2.62037E-17 | up | down | down |
| miR-101 | 1.39 | 0.38 | 1.3577E-11 | up | down | ns |
| miR-374 | 3.70 | 1.62 | 8.09259E-11 | up | ns | ns |
| **miR-200b** | 0.24 | 0.02 | 8.09259E-11 | **up** | **up** | **up** |
| miR-133a | 2.55 | 0.85 | 1.77528E-10 | up | down | down |
| miR-186 | 3.12 | 1.31 | 1.44139E-09 | up | ns | ns |
| **miR-193b** | 1.74 | 0.64 | 4.51472E-09 | **up** | **up** | **up** |
| **miR-20b** | 2.60 | 0.88 | 6.59851E-09 | **up** | ns | **up** |
| miR-140 | 2.34 | 0.96 | 6.59851E-09 | up | down | ns |
| miR-143 | 1.51 | 0.60 | 6.59851E-09 | up | down | down |
| **miR-19a** | 4.49 | 2.38 | 7.85773E-09 | **up** | **up** | ns |
| miR-195 | 3.75 | 1.76 | 9.70587E-09 | up | down | down |
| miR-140-3p | 0.89 | 0.25 | 9.70587E-09 | up | down | NA |
| let-7b | 5.49 | 3.18 | 1.34612E-08 | up | down | ns |
| **miR-16** | 10.03 | 7.46 | 2.41729E-08 | **up** | down | **up** |
| miR-29a | 2.04 | 0.86 | 2.52565E-08 | up | ns | ns |
| **miR-200c** | 0.65 | 0.17 | 2.53174E-08 | up | **up** | **up** |
| **miR-17** | 8.97 | 6.93 | 3.43933E-08 | **up** | ns | **up** |
| miR-191 | 7.17 | 4.99 | 3.94415E-08 | up | ns | ns |
| miR-340 | 0.33 | 0.09 | 3.94415E-08 | up | ns | ns |
| **miR-93** | 4.41 | 2.29 | 3.95738E-08 | **up** | ns | **up** |
| miR-26b | 4.82 | 2.44 | 8.07918E-08 | up | down | up |
| **miR-106a** | 8.77 | 6.30 | 1.87999E-07 | **up** | ns | **up** |
| miR-365 | 0.23 | 0.02 | 2.22035E-07 | up | ns | ns |
| miR-145 | 0.81 | 0.27 | 3.25968E-07 | up | down | down |
| miR-20a | 8.75 | 6.45 | 6.18902E-07 | up | ns | ns |
| **miR-301** | 1.92 | 0.83 | 1.0864E-06 | **up** | **up** | **up** |
| miR-222 | 4.94 | 3.41 | 1.84847E-06 | up | down | up |
| miR-151-3p | 1.06 | 0.35 | 4.21132E-06 | up | ns | ns |
| miR-142-5p | 0.54 | 0.20 | 4.39725E-06 | up | ns | ns |
| miR-451 | 9.81 | 7.57 | 4.71947E-06 | up | down | down |
| **miR-106b** | 4.85 | 3.40 | 5.50091E-06 | up | **up** | **up** |
| miR-532-3p | 0.79 | 0.29 | 7.89379E-06 | up | down | NA |
| miR-185 | 2.52 | 1.26 | 9.7517E-06 | up | down | up |
| **miR-19b** | 9.29 | 7.24 | 1.41989E-05 | up | ns | **up** |
| **let-7g** | 3.59 | 2.42 | 1.76868E-05 | up | ns | **up** |
| miR-574-3p | 2.79 | 1.56 | 2.13278E-05 | up | ns | NA |
| **miR-215** | 2.91 | 1.67 | 2.29278E-05 | up | **up** | ns |
| miR-126 | 7.06 | 5.62 | 2.54247E-05 | up | down | down |
| **miR-25** | 4.04 | 2.67 | 4.69252E-05 | up | ns | **up** |
| miR-22* | 0.43 | 0.21 | 0.000313258 | up | ns | NA |
| miR-26a | 5.04 | 3.70 | 0.000407577 | up | down | ns |
| **miR-331** | 2.88 | 2.06 | 0.000415398 | up | ns | **up** |
| **miR-141** | 0.47 | 0.13 | 0.000569015 | **up** | **up** | **up** |
| let-7e | 4.16 | 3.14 | 0.000606613 | up | down | down |
| **miR-744** | 1.19 | 0.61 | 0.000667911 | **up** | **up** | NA |
| miR-345 | 0.74 | 0.41 | 0.001284154 | up | ns | ns |
| **miR-758** | 11.20 | 4.40 | 0.001973577 | **up** | **up** | NA |
| miR-223 | 12.37 | 10.90 | 0.002223734 | up | down | ns |
| **miR-93*** | 0.75 | 0.38 | 0.003588602 | **up** | **up** | NA |
| miR-425-5p | 3.78 | 2.72 | 0.003837613 | up | ns | ns |
| miR-324-5p | 1.05 | 0.60 | 0.004120631 | up | ns | ns |
| miR-197 | 3.59 | 2.83 | 0.004860341 | up | ns | ns |
| miR-296 | 1.19 | 0.66 | 0.005192843 | up | ns | ns |
| miR-342-3p | 3.39 | 2.43 | 0.005976843 | up | ns | down |
| miR-339-3p | 0.32 | 0.08 | 0.006451972 | up | ns | NA |
| **miR-24** | 7.01 | 6.08 | 0.007079054 | up | ns | **up** |
| miR-532 | 2.80 | 1.92 | 0.007713229 | up | down | NA |
| miR-133b | 0.32 | 0.11 | 0.007713229 | up | down | down |
| miR-320 | 5.91 | 5.19 | 0.009199304 | up | ns | ns |
| miR-99b* | 1.16 | 2.22 | 0.004141788 | down | ns | NA |
| miR-212 | 1.08 | 2.25 | 0.00226506 | down | up | ns |
| miR-661 | 1.78 | 3.07 | 0.000183617 | down | ns | NA |
| miR-646 | 9.66 | 11.59 | 0.009503205 | down | NA | NA |
| miR-203 | 0.75 | 2.70 | 0.000571435 | down | ns | up |
| miR-648 | 1.41 | 3.61 | 8.17852E-05 | down | NA | NA |
| miR-643 | 7.85 | 10.13 | 0.001106467 | down | NA | NA |
| miR-886-3p | 1.55 | 4.12 | 0.009339206 | down | up | NA |
| miR-1243 | 19.04 | 21.70 | 0.00036593 | down | ns | NA |
| miR-302c* | 8.10 | 10.79 | 0.004120631 | **down** | NA | **down** |
| miR-659 | 2.51 | 5.20 | 0.00062641 | down | up | NA |
| miR-518f | 15.19 | 18.39 | 0.003116176 | down | ns | ns |
| miR-520e | 8.28 | 11.74 | 0.000359152 | down | up | ns |
| miR-1183 | 11.38 | 14.85 | 0.000352128 | down | ns | NA |
| miR-135b* | 2.10 | 5.79 | 0.00047552 | down | ns | ns |
| miR-367 | 5.53 | 9.35 | 0.000667911 | down | up | ns |
| miR-34a | 3.88 | 7.88 | 4.69252E-05 | down | ns | ns |
| miR-601 | 5.12 | 9.22 | 0.00864536 | down | up | NA |
| miR-302a | 5.03 | 9.17 | 4.21132E-06 | down | up | ns |
| miR-551b | 10.17 | 14.42 | 2.32667E-08 | **down** | **down** | NA |
| **miR-206** | 1.11 | 5.58 | 0.00017413 | **down** | **down** | **down** |
| miR-302c | 9.82 | 14.31 | 0.000115697 | down | NA | ns |
| miR-136 | 12.04 | 16.55 | 0.007651159 | down | ns | ns |
| miR-194 | 2.13 | 6.78 | 0.005880776 | down | up | ns |
| miR-155 | 3.74 | 8.47 | 0.003176074 | down | up | up |
| miR-572 | 6.42 | 11.20 | 0.006981808 | down | ns | ns |
| miR-324-3p | 6.60 | 11.81 | 0.007589587 | **down** | **down** | ns |
| miR-454 | 2.90 | 8.92 | 0.002688825 | down | ns | NA |
| miR-708 | 5.00 | 11.22 | 0.000189801 | down | up | ns |
| miR-491 | 2.69 | 9.69 | 0.00092377 | down | ns | ns |
| miR-34a* | 3.17 | 12.29 | 4.85906E-05 | down | ns | NA |

**Supplementary Table S3.** Pathway enrichment analysis using DIANA miRPath v2.0 based upon predicted targeted genes (microT threshold 0.9) of 5 miRNAs significantly upregulated in NSCLC tissues and serum. Statistically significant (p<0.001) pathways comprising ≥ 5 genes are shown. Relevant pathways in lung cancer or lung development are depicted in bold.

| **KEGG pathway (*p*<0.001)** | **# genes** | **# miRNAs** |
| --- | --- | --- |
| **MAPK signaling pathway** | 33 | 3 |
| **PI3K-Akt signaling pathway** | 29 | 3 |
| Endocytosis | 25 | 2 |
| **Neurotrophin signaling pathway** | 22 | 3 |
| Axon guidance | 20 | 3 |
| **Focal adhesion** | 14 | 2 |
| Pathways in cancer | 13 | 1 |
| Renal cell carcinoma | 12 | 3 |
| HTLV-I infection | 12 | 2 |
| Regulation of actin cytoskeleton | 11 | 2 |
| **ErbB signaling pathway** | 10 | 2 |
| Ubiquitin mediated proteolysis | 9 | 1 |
| Glioma | 8 | 3 |
| **HIF-1 signaling pathway** | 8 | 2 |
| Porphyrin and chlorophyll metabolism | 8 | 1 |
| Fc gamma R-mediated phagocytosis | 8 | 2 |
| Starch and sucrose metabolism | 8 | 1 |
| **Steroid hormone biosynthesis** | 7 | 1 |
| Lysine degradation | 7 | 3 |
| Ascorbate and aldarate metabolism | 7 | 1 |
| Pentose and glucuronate interconversions | 7 | 1 |
| Insulin signaling pathway | 7 | 1 |
| mTOR signaling pathway | 6 | 2 |
| Phosphatidylinositol signaling system | 6 | 2 |
| Inositol phosphate metabolism | 6 | 2 |
| Non-small cell lung cancer | 5 | 2 |
| GnRH signaling pathway | 5 | 2 |
| Chronic myeloid leukemia | 5 | 1 |

**Supplementary Figures.**

**A Novel Serum 4-microRNA Signature for Lung Cancer Detection**

Ernest Nadal M.D., Anna Truini M.S., Asuka Nakata Ph.D., Jules Lin M.D., Rishindra M. Reddy M.D., Andrew C. Chang M.D., Nithya Ramnath M.D., Noriko Gotoh Ph.D., David G. Beer Ph.D. and Guoan Chen M.D., Ph.D.

**Supplementary Figure S1.** Raw Ct value distribution of U6 snRNA in the validation set (n = 60).


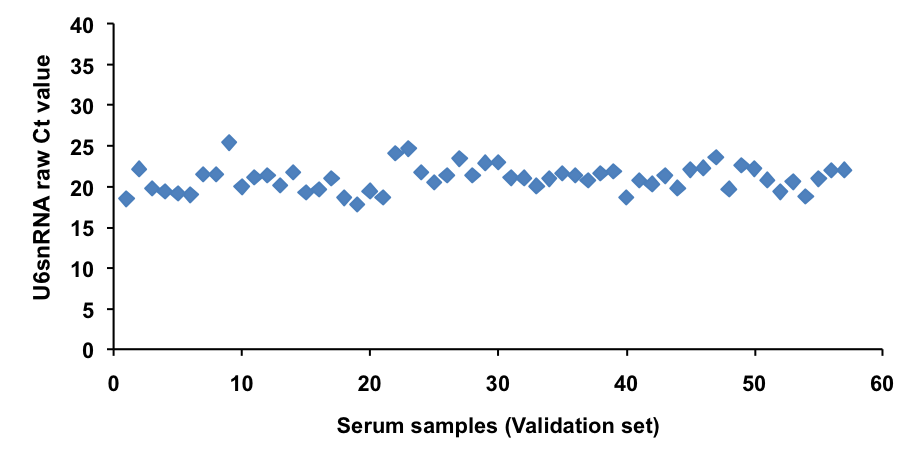


**Supplementary Figure S2.** **.** Relative expression of miR-141, miR193b, miR-200b and miR-301a across a large collection of human cancer cell lines (NCI-60). Extracted from GSE26375. Lung cancer cell lines expressed relatively high levels of those miRNAs.


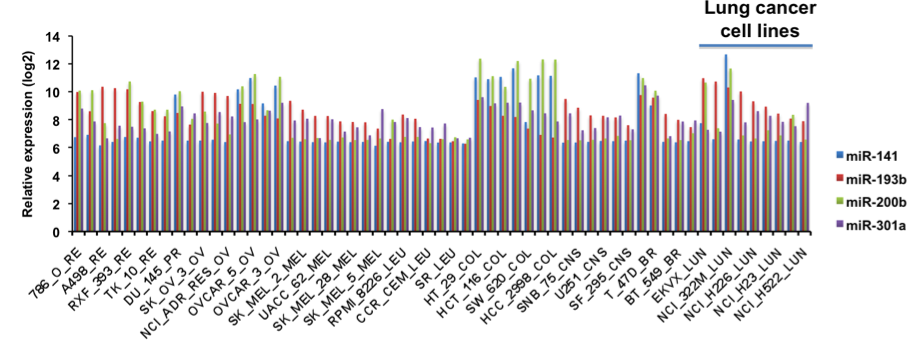


**Supplementary Figure S3.** Kaplan-Meyer plots of Disease-free survival according to the expression level of serum miR-193b, miR-301, miR-141 and miR-200b in the training set. Red line ≥ median (n=35); blue line < median (n=35).


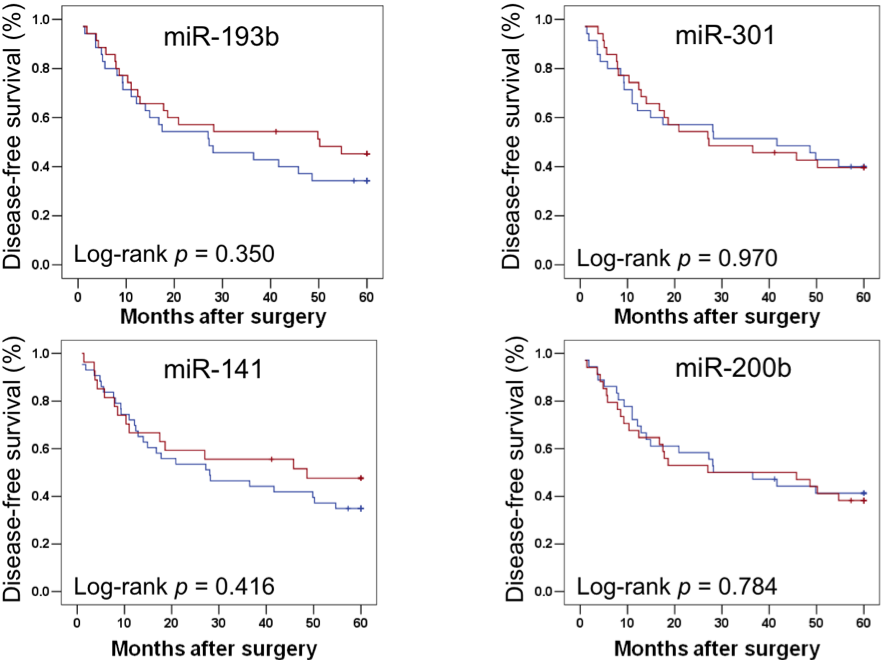

Supplement: Supplementary Information [file srep12464-s1.doc]
